# Rethinking ovary preservation by adnexal torsion reversal in adolescents: a case of delayed diagnosis

**DOI:** 10.1186/s12905-022-02013-4

**Published:** 2022-10-24

**Authors:** Yue He, Chen Ji, Xiao-Chang Shen, Ke-Xin Zhang, Yu-Mei Wu, Yan Wang

**Affiliations:** Department of Gynecological Oncology, Beijing Obstetrics and Gynecology Hospital, Capital Medical University. Beijing Maternal and Child Health Care Hospital, Dongcheng District, 17 Qihelou Street, Beijing, 100006 China

**Keywords:** Ovary preservation, Delayed diagnosis, Adnexal torsion, Adolescent, Case report

## Abstract

**Background:**

This article discusses the management of an adolescent woman with a delayed diagnosis of adnexal torsion (AT) whose ovaries were successfully preserved.

**Case presentation:**

The patient was a 14-year-old female teen admitted with the chief complaint of lower abdominal pain for 3 days and worsening pain for 2 days. Magnetic resonance imaging suggested a high possibility of torsion in the anterosuperior uterine mass and was accompanied by severe ovarian edema, bleeding, and enlargement. Intraoperatively, the left fallopian tube was characterized by thickening and torsion and appeared blackish purple. The left fallopian tube paraovarian cyst was about 20 cm in size, and the left adnexa was twisted 1080° along the left infundibulopelvic ligament (suspensory ligament of the left ovary). The left ovary appeared blackish purple, with an enlarged diameter of about 10 cm. At the request and with the informed consent of the patient’s parents, we preserved the left ovary and removed the left fallopian tube. The results of the endocrine, ultrasound, and tumor marker tests were normal 1 month after surgery. Follicles and blood flow signals seen in ultrasound examinations indirectly proved the successful preservation of the left ovary in the follow-up.

**Conclusions:**

Our attempt to preserve the ovaries in an adolescent with a delayed diagnosis of AT was successful.

## Background

Adnexal torsion (AT) is an anatomical translocation of the ovaries and/or fallopian tubes along the axis of the infundibulopelvic ligament and the suspensory ligaments of the ovaries, resulting in partial or total obstruction of the blood supply to the ovary. It is ranked as the fifth most common acute abdomen in gynecological practice [1, 2]. AT can occur in women of any age, but it mostly occurs in women of childbearing age, followed by children and adolescents, with 30% being girls aged < 20 years, and 46% of cases in adolescents only had torsion of the ovary, without ovarian cysts [2]. The most common clinical presentation of AT is the sudden onset of localized lower abdominal pain with or without nausea and vomiting. Ultrasonography, computed tomography (CT), magnetic resonance imaging (MRI), and other examinations have auxiliary significance in diagnosing AT.

With improvements in the understanding of AT, several studies have shown that recovery of ovarian function is possible after torsion release, regardless of the visual appearance of the AT [1, 3]. Therefore, once AT is suspected, prompt diagnostic surgery should be performed to release the torsion and preserve ovarian function and fertility as much as possible. At present, there is a new understanding of the early diagnosis of AT and the choice of surgical approach. This article discusses the management of an adolescent woman with a delayed diagnosis of AT who had successful ovarian preservation.

## Case presentation

The patient was a 14-year-old female teen admitted with a chief complaint of lower abdominal pain for 3 days, which exacerbated for 2 days. Menarche had occurred at 10 years of age, the patient had regular menstruation, and her last menstrual period was on January 10, 2022. Three days before admission, she experienced spontaneous, dull pain in the lower abdomen after changing posture. Ultrasonography conducted in another hospital showed a large cyst on the right side of the pelvis measuring 19.6 × 9.6 cm. Moderate to low echogenicity with an area of about 11.5 × 6.3 cm was observed in the mid-upper pelvic cavity; she sought medical attention at our hospital. Two days before admission (the date of admission was January 10, 2022), she experienced increased abdominal pain with hypothermia and vomiting, but dizziness, headache, palpitations, and shortness of breath were absent. She did not undergo diagnosis or treatment for personal reasons and underwent consultation at our department for further pelvic examination. A gynecological examination showed abdominal distension, a palpable mass, positive tenderness, and rebound tenderness. She denied any history of chronic diseases, drug allergies, surgery, or blood transfusions.

At the time of admission, her temperature, blood pressure, and pulse rate were 37.7 °C, 140–145/80–90 mmHg, and 80–90 beats/minute, respectively. On physical examination, abdominal distension, fixed lower abdominal tenderness, rebound tenderness, and shifting dullness were negative. Gynecological examination (anal examination) revealed a mass that could be palpated at the pelvis, positive and rebound tenderness, and smooth rectal mucosa.

The ancillary examinations were as follows:

1. Routine blood test:white blood cells (WBC), 16.88 × 10^9^/L [4 × 10^9^–10 × 10^9^]; hemoglobin (HGB), 97 [110–150] g/L; and platelets (PLT), 227 × 10^9^/L [100 × 10^9^–300 × 10^9^].
2. Tumor markers:human epididymis protein 4 (HE4), 40.44 [0–92] pmol/L; alpha fetoprotein (AFP), < 0.61 [0–7] ng/mL; carbohydrate antigen 199 (CA199), 3.84 [0–39] U/mL; and CA125, 66.97 [0–35] U/mL.
3. Endocrine tests: follicle-stimulating hormone (FSH), 4.06 [1.50–9.10] IU/L; estrogen 2 (E2), 21.3 [52.80–214.20] pg/mL; progestogen (P), 0.289 [4.44–28.03] ng/mL; luteinizing hormone (LH), 5.5 [0.50–16.90] IU/L; testosterone (T), 2.52 [0.31–1.66] nmol/L; and anti-Mullerian hormone (AMH), 6.39 [0.24–11.78] ng/mL.
4. Enhanced MRI: The left uterine horn was stretched upward, the uterine fundus was approximately 4.3 × 3.0 × 5.1 cm in size, and a solid mass was seen in the anterosuperior uterus, approximately 10.3 × 6.4 × 11.5 cm in size, showing iso-T1, a short T2 predominantly mixed signal, a flocculent long T2 signal, and a low signal on DWI, with small cystic foci scattered in the periphery, non-enhanced or mildly heterogeneous, strengthening on enhanced MRI, and another larger cystic foci with long T1 and long T2 signals that were connected with the above solid mass, measuring approximately 19.2 × 9.9 × 14.2 cm, with a thick and enhanced cystic wall on enhanced MRI, and pelvic effusion. No abnormally enlarged lymph nodes were seen in the pelvis and bilateral inguinal regions. Diagnostic conclusions included a solid mass in the anterosuperior uterus and an enlarged left ovary; the right upper cystic mass was considered a left ovarian cystic mass, and the possibility of torsion in the left ovary and its cystic mass was high and accompanied by severe ovarian edema, bleeding, enlargement, and pelvic effusion.

**Fig. 1:**
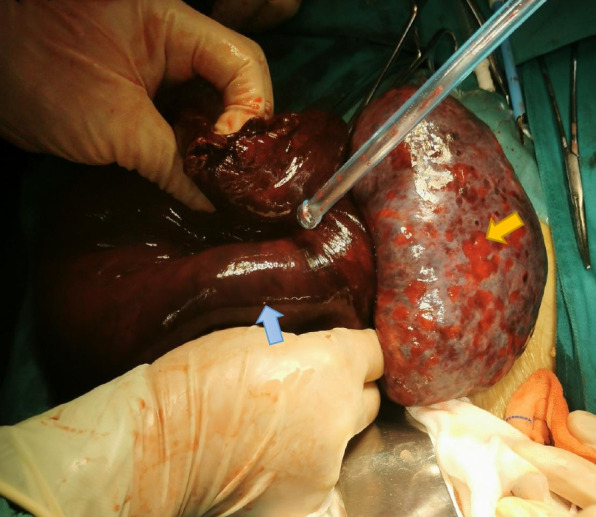
Intraoperative condition. The left fallopian tube was thickened, twisted, and blackish purple. The left fallopian tube paraovarian cyst was about 20 cm in size (blue arrow), the left adnexa was twisted 1080° along the left infundibulopelvic ligament–suspensory ligament of the left ovary (high tension), and the left ovary was blackish purple with an enlarged diameter of about 10 + cm (yellow arrow). The color of the left ovary was slightly restored after reduction of the left adnexa, whereas the left fallopian tube remained blackish purple

The intraoperative condition (Fig. 1) was as follows: small amounts of cool yellowish fluid were found in the pelvis, the left fallopian tube was characterized by thickening and torsion and appeared blackish purple, the left fallopian tube paraovarian cyst was about 20 cm in size, the left adnexa twisted 1080° along the left infundibulopelvic ligament (suspensory ligament of the left ovary) (high tension), and the left ovary was blackish purple with an enlarged diameter of about 10 cm. The color of the left ovary was slightly restored after the reduction of the left adnexa, and there was no obvious cyst in the ovary, while the left fallopian tube remained blackish purple.

**Fig. 2:**
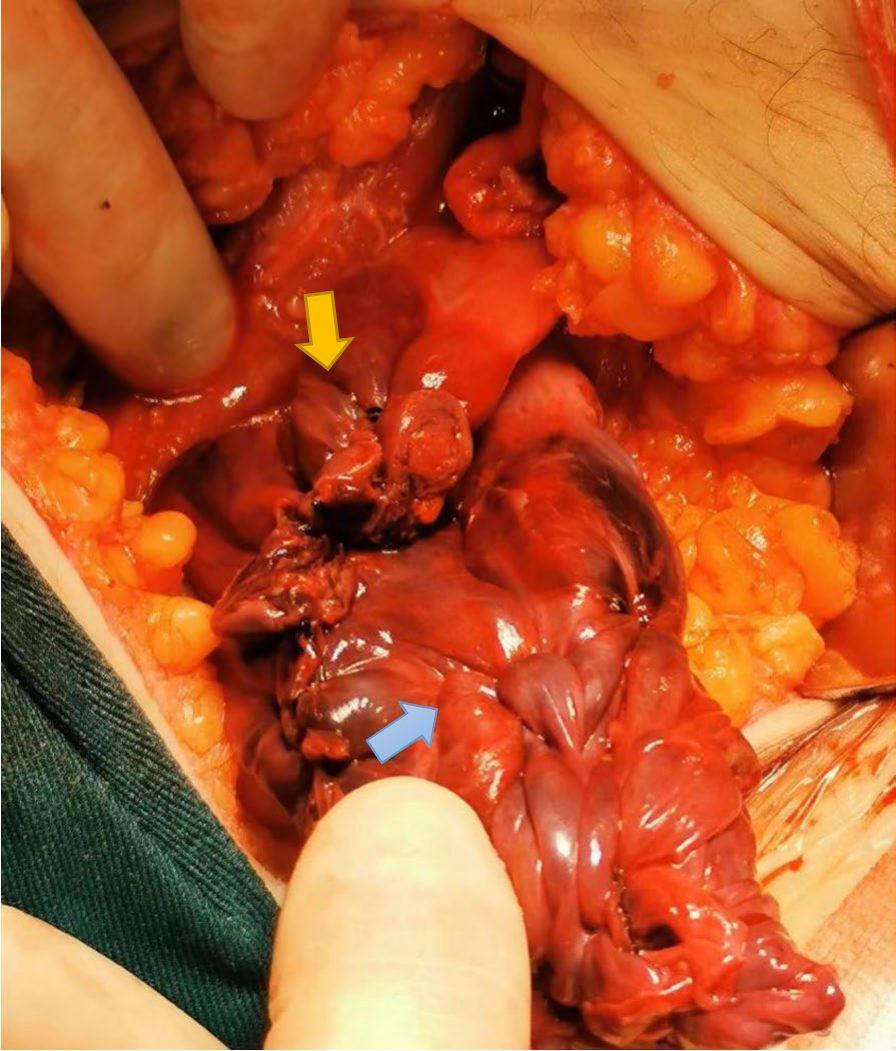
Postoperative condition. The left ovary was preserved (blue arrow), and the left fallopian tube was removed (yellow arrow), and at the same time, blood clot cleaning of the left ovary was performed

The patient’s parents informed us that the patient was young and had a strong willingness to preserve her ovaries and that she could be followed-up closely. They were informed with the following details: there was a possibility of re-torsion if the ovaries were preserved; the blood supply to the ovaries was poor; surgery might be needed if necrosis occurred; the left fallopian tube was twisted and hypertrophied, had poor blood supply, and had lost its normal form and function; and re-torsion, hydrosalpinx, and ectopic pregnancy may occur if the left fallopian tube was preserved. Based on the latest consensus, left ovary preservation was considered, but the recommendation was to resect the left fallopian tube. The patient’s family expressed their understanding. At the request and with the informed consent of the patient’s parents, we preserved the left ovary, removed the left fallopian tube, and performed blood clot cleaning of the left ovary (Fig. 2). For further treatment according to the specific postoperative conditions and pathology, we did not perform a frozen section because there was no evidence of malignancy observable by the naked eye.

On day 1 after surgery, she had a transient temperature increase of 39 °C, which later spontaneously decreased to normal, with good gas expulsion and defecation.

Reexamination results were as follows: (1) coagulation: fibrinogen (FIB), 6.7 [1.8–4] g/L; activated partial thromboplastin time (APTT), 28.4 [23.3–32.5] s; thrombin time (TT), 14.3 [14–21]s; international normalized ratio (INR), 1.030 [0.8–4]; and D-dimer (D-D), 5.13 [0–0.55] mg/L. (2) Routine blood tests showed the following: WBC, 10.35 × 10^9^/L [4 × 10^9^–10 × 10^9^]; HGB, 95 [110–150] g/L; and PLT, 365 × 10^9^/L [100 × 10^9^–300 × 10^9^].

A postoperative pathology test suggested a left fallopian tube paraovarian cyst with fibrocystic wall-like tissue in the left ovary, extensive congestion, and hemorrhage, and no apparent lining epithelium, consistent with torsional pathological changes.

One month after surgery, the results of the endocrine, ultrasound, and tumor marker tests were normal, and menstruation was normal. The results of the endocrine tests were as follows: FSH, 7.35 [1.50–9.10] IU/L; E2, 35.7 [52.80–214.20] pg/mL; P, 0.223 [4.44–28.03] ng/mL; LH, 17.0 [0.50–16.90] IU/L; T, 1.680 [0.31–1.66] nmol/L; and AMH, 4.14 [0.24–11.78] ng/mL. Ultrasound:The left ovary was 5 cm in the maximum longitude size, with uneven echo. Multiple cysts (13 cysts/plane) were seen, and the diameter of the larger cysts was 0.7 cm. Blood flow signals could be seen in Color Doppler Flow Imaging (CDFI) ovary, Resistance Index (RI) = 0.77, Pulse Index (PI) = 1.97 (yellow arrow). The follicles and blood flow signals revealed by ultrasound, indirectly proved the successful preservation of the left ovary.

## Discussion

### Management of AT

In recent years, early diagnosis and standardized management of AT have received attention from Chinese and overseas experts. Surgical exploration is considered the standard method to confirm the diagnosis of AT. The incidence of AT in women aged 1–20 years was 5/100,000 in a US study [2], and the results of a Korean study were consistent with that of the US findings [3]. Studies have reported [2, 4] that the incidence of malignant prepubertal adolescent ovarian cysts is 0.5–4%, and the incidence of malignancy in children with ovarian cysts and AT is only 1.8%. The American College of Obstetricians and Gynecologists (ACOG) [2] and the Chinese Medical Doctors’ Association [5] developed an expert consensus for AT in American and Chinese women in 2019 and 2020, respectively. Both consensuses provide detailed summary norms and guidelines on AT regarding clinical presentation, differential diagnosis, treatment, and follow-up. Two expert consensuses emphasize that age is a critical factor for selecting torsional reduction or adnexal resection and that premenopausal patients should undergo routine AT reduction with preservation of the affected adnexa, even if the ovary is purplish black and necrosis is suspected when observed intraoperatively by the naked eye. In some cases, the ovary’s color does not return to normal immediately after reduction but takes 36 h to do so after torsion release [2]. Adnexal resection should be considered for postmenopausal patients whose ovarian function has severely reduced and whose adnexal masses have a relatively high chance of malignancy.

The guidelines of the Royal College of Obstetricians and Gynecologists suggest that in premenopausal women, adnexal masses > 7 cm in diameter have an increased risk of rupture and therefore require elective surgical management [6]. Moreover, the mean diameter of the adnexa (encapsulated mass) was 6.2 cm higher in patients with AT than the mean diameter (4.2 cm) of the adnexa (including the mass) in those without torsion [OR 95% CI 2.88 (1.15–7.21)]; hence, the diameter of the adnexa (including the mass) being > 6 cm was considered the surgery threshold [6].

### Concerns regarding preserved ovaries after ovarian torsion

Most scholars view that the intraoperative finding of a darkened ovary means that it is necrotic, AT reduction increases the incidence of thromboembolic events, and surgical treatment is mostly performed by resection of the affected adnexa, which has led to many unnecessary adnexal resections. The reported incidence of pulmonary embolism after AT was only 0.2%, and AT reduction did not increase the incidence of thromboembolic events [7]. The intraoperative coloration of the ovaries under the naked eye is also not a reliable basis for determining whether the ovary is necrotic [8, 9]. Several studies have shown that an ovary that was found to be severely ischemic during surgery can return to normal function after reduction [10]. However, in clinical practice, when we see the black and purple appendages after torsion and the infundibulopelvic ligament is filled with a purplish blood clot, any doctor may find it difficult to reduce and preserve the appendage. Although the risk of thrombosis is very low, it will seriously endanger the lives of young women if it occurs. Further validation is required to see if the ovarian function is retained after preservation. The only thing that can help determine the recovery of the ovaries is their size and ovulation under ultrasound monitoring—absent Doppler flow is a sign of ovarian necrosis. Clinical correlation between ultrasonography findings and the patient’s symptoms makes the diagnosis of AT more accurate and prompt [11]. However, it is unknown whether the reduced ovary is congested, edematous, or even necrotic due to ischemia, which will have more serious consequences if there is secondary infection or rejection.

Another concern is that the preserved ovaries still have the potential for future re-torsion. In addition, whether or not to perform ovarian fixation still needs to be determined in a study with a large sample size. In our patient, we unfortunately removed a unilateral fallopian tube that was edematous, severely deformed, and possibly severely impaired in function; however, fortunately, the ovary was preserved. We observed normal ovarian structures and ovulation under ultrasonography during the follow-up monitoring, indirectly proving the feasibility that even purplish-black ovaries should be preserved. Moreover, we realized that based on the pathological findings, it was not difficult to guess that this young patient could have had a comorbid paraovarian cyst that had twisted after activity, resulting in ovarian congestion, edema, and even ischemic necrosis, but the ovary itself did not have any lesion. The function of the twisted ovary could be preserved if we performed reduction alone, but postoperative clot absorption would increase the risk of fever or even infection, and the enlarged appendage would lead to re-torsion. The follow-up results proved that this method could increase the success of ovary preservation while partially retaining the function of the left ovary. In the literature, the overall incidence of recurrence of AT (rAT) in female teens was reported to be low, approximately 2–12% [8]. A study reported [12] that three risk factors are associated with AT: enlarged adnexa, preservation of the ovaries in the previous surgery, and in vitro fertilization (IVF) treatment. Cohort studies have shown that the incidence of adnexal re-torsion without the combination of these high-risk factors, with one, two, or three high-risk factors, was 44.4, 67.9, 82.9, and 100%, respectively. Women who are at high risk for these factors should be closely monitored, especially if they require IVF, and, if necessary, laparoscopic exploration should be performed.

In our patient, the adnexal cyst was seen intraoperatively to be a paraovarian cyst and was large enough to cause overall torsion of the left adnexa, and the ovary was edematous, congested, and even necrotic due to progressive ischemia. We did not see a solid mass during intraoperative dissection of the ovary; hence, we removed the left ovarian necrosis and clots, and a drainage tube was inserted into the pelvis, which reduced the probability of postoperative infection and fever, reduced the size of the ovary, and reduced the likelihood of recurrence of adnexal re-torsion. If the female teen had undergone early detection and treatment of the paraovarian cyst, she might not have been in such a passive situation, with the left fallopian tube necrotic and severely twisted, leading to its removal and the left ovarian function being severely affected. Therefore, we recommend performing laparoscopic exploration in female teens who are found to have adnexal cysts, if the adnexal diameter (including the mass) is > 6 cm, and persists for 3 months (excluding physiological ones), or if it is accompanied by discomfort such as dull pain in the lower abdomen with adnexal cysts persistence. Although the incidence is extremely low, it indirectly suggests that paraovarian cysts should not be ignored. Periovarian cysts and surgery should be recommended if there is intraoperative or imaging evidence of persistent and enlarged masses.

The following reasons are summarized in the case of our adolescent female teen who successfully preserved her ovaries: (1) The time to torsion was about 72 h, and we found that the AT was quickly treated surgically to give adequate time for the recovery of the torsioned adnexa. The longer the interval between the onset of AT and surgical treatment, the greater the likelihood of ovarian damage. Therefore, emergency surgical intervention is required once AT is diagnosed. The time between the disruption of the adnexal blood supply and irreversible ovarian damage is unclear; however, some studies have suggested that ovarian function may still be restored beyond 72 h of AT, but most believe that ovarian function begins to decline dramatically after 48–72 h [9]. Early and rapid surgical intervention can better protect ovarian function and fertility. (2) The patient was young in good physical condition, had an abundant blood supply to the ovaries, and recovered quickly. (3) The patient’s family cooperated, and the patient received follow-up and a second surgery, giving the surgeon confidence and creating a chance to preserve the ovary successfully. (4) We administered anti-inflammatory and anti-thrombotic treatment before and after the surgery to prevent infection and thromboembolism.

Currently, there are no guidelines on the prevention of AT for female teens in the expert AT consensus. For adolescent women who have had their menarche before aged < 20 years, parents and schools often neglect gynecological examinations during routine physical examinations because they have not yet started becoming sexually active, and because there are more activities and sports at this age. Cysts also grow gradually with age; hence, they are often detected only when they are huge and affect patients’ lives or when they are found to have reached an acute stage such as torsion or rupture. The symptoms are also atypical and are easily missed and misdiagnosed. Therefore, prevention is more important than the treatment of children and female teens, and it is recommended to increase gynecological ultrasound screenings every year. Moreover, physiological health-related scientific knowledge should be imparted so that girls will not be embarrassed if they develop abdominal pain and discomfort and will instead seek timely medical attention.

### Possible surgical method of ovarian torsion

Regarding the choice of surgical approach in the consensus, laparoscopic exploratory surgery was suggested to be used as the first choice. However, the ovary and cyst will increase dramatically after adnexal mass necrosis and torsion. Often, the adnexal mass is about 20 cm in diameter, and the thickening of the infundibulopelvic is often accompanied by thrombosis, which might cause significant difficulties for laparoscopic surgery. The surrounding structures cannot be seen clearly, and it is easy to cause collateral damage. Whether laparoscopic surgery has absolute advantages over small-incision open surgery needs to be examined, but laparoscopic surgery is a good option if used only as an experimental procedure to provide an opportunity for adnexal reduction. Even if adnexal masses with diameters > 10 cm have a higher chance of intraoperative spillage, this does not implicate an a priori need for proceeding with open surgery, particularly when the risk of malignancy is low. The presence of ascites and the size of the lesion associated with a high level of CA 125 affected the correct assessment of the risk of malignancy, exposing the patient to overtreatment [13]. Considering the confirmed advantage in operative time and intraoperative blood loss [14], current evidence suggests performing laparoscopic ovarian detorsion; although this procedure is acceptable, we must keep in mind that ischemia/reperfusion (I/R) injury can extend and worsen ischemic and necrotic damage [15].

Our patient underwent emergency surgery, and the preoperative investigation of the adnexal mass revealed that the size of the mass reached the level of the umbilicus, the mass was huge, and the patient was considered to have a true cyst present with an unknown multi-compartment-like nature; hence, a longitudinal small-incision open surgery was performed. However, in retrospect, it may have been feasible if laparoscopic exploration had been performed at that time, the left fallopian tube was resected after reduction, and the left ovary was preserved. Ovarian function can be observed after waiting 6–8 weeks to decide on the follow-up treatment.

## Conclusions

We experienced several cases of ovarian cyst torsion in adolescent women in the last year. After the reduction in adolescents and children, the symptoms are not typical, and many patients are found to have purplish-black ovaries. Some of these affected girls lose unilateral adnexa, which is deplorable. Our attempt to preserve the ovaries in adolescents with delayed diagnosis of AT was successful in this case and further confirmed the validity of the current consensus on AT in the United States and China. We hope that in future guidelines and consensus improvement, scientific education on physiological anatomy and physiological hygiene of adolescents can be increased and the risk management-related issues such as preventive measures and response to the onset of infection and thrombosis should be given attention during postoperative pain management.

## Data Availability

All data generated or analyzed during this study are included in this published article.

## Abbreviations

AFP: Alpha fetoprotein
AMH: Anti-Mullerian hormone
AT: Adnexal torsion
CA 125: Carbohydrate antigen 125
CT: Computed tomography
D-D: D-Dimer
E2: Estrogen 2
FSH: Follicle-stimulating hormone
FIB: Fibrinogen
HGB: Hemoglobin
LH: Luteinizing hormone
MRI: Magnetic resonance imaging
P: Progestogen
PLT: Platelets
T: Testosterone
WBC: White blood cell
